# Emergence of azithromycin and ciprofloxacin non-susceptible genotype 4.2.2 *S*. Typhi in Fiji

**DOI:** 10.1128/aac.00588-25

**Published:** 2025-09-22

**Authors:** Ashwini Vinod, Andrew J. Hayes, Yogeshni Chandra, Sonika Kaajal Nair, Tashyam Pillay, Farieya Fayza Khan, Shalini Goundar, Luse Dulaki Kalou, Khushnuma Khan, Maria Borua Anise, Mary Valcanis, Xinwei Ruan, Shalini Singh, Jessica Barnden, Raphaël M. Zellweger, Alumita Vuakanisakea, Kylie Hui, Lisa J. Ioannidis, Kim Mulholland, Richard A. Strugnell, Benjamin P. Howden, Mark R. Davies, Aneley Getahun Strobel

**Affiliations:** 1 Lautoka Hospital, Aspen Medical Health Care Fiji limited, Lautoka, Fiji; 2 Department of Microbiology and Immunology, The University of Melbourne at the Peter Doherty Institute for Infection and Immunity 2281, Melbourne, Australia; 3 Microbiological Diagnostic Unit Public Health Laboratory, Department of Microbiology and Immunology, The University of Melbourne at the Peter Doherty Institute for Infection and Immunity 2281, Melbourne, Australia; 4 Fiji Centre for Disease Control, Ministry of Health and Medical Services, Suva, Fiji; 5 International Vaccine Institute, Seoul, South Korea; 6 Live and Learn Environmental Education, Suva, Fiji; 7 WHO Collaborating Centre for Antimicrobial Resistance, The University of Melbourne at the Peter Doherty Institute for Infection and Immunity 2281, Melbourne, Australia; 8 Centre for Pathogen Genomics, The University of Melbourne 2281, Melbourne, Australia; 9 Murdoch Childrens Research Institute, Melbourne, Australia; 10 London School of Hygiene and Tropical Medicine 4906, London, United Kingdom; Columbia University Irving Medical Center, New York, New York, USA

**Keywords:** Typhoid, *Salmonella* Typhi, Oceania, Fiji, Ciprofloxacin resistance, AcrB mutation, antimicrobial resistance, genomic epidemiology

## LETTER

Typhoid is a systemic infection caused by the bacterium *Salmonella enterica* subspecies *enterica* serovar Typhi (*S*. Typhi) (1, 2). Typhoid is endemic in the island nation of Fiji in the South Pacific and mainly reported in indigenous Fijians and males, with a peak age of incidence between 15 and 29 years (3–5). *S*. Typhi isolates in Fiji are associated with a single evolutionary-related endemic genotype (4.2) comprised of two lineages—4.2.1 and 4.2.2, and antimicrobial resistance (AMR) is uncommon (5–9). In this study, we conduct a retrospective genomic epidemiology study of *S*. Typhi in Fiji and describe the presence of extant *S*. Typhi isolates with multiple chromosomal mutations (GyrA S83F and AcrB efflux pump R717Q) altering susceptibility to ciprofloxacin and azithromycin. These resistance findings have not previously been reported in the genotype 4.2 and were not associated with international strain importation events.

Our primary study site was the Western Division of Fiji with 337,041 inhabitants accounting for 38.1% of the total population of Fiji (10). The Lautoka hospital is the main tertiary hospital in the division and is a referral center for six subdivisional hospitals (Rakiraki, Tavua, Ba, Lautoka/Yasawa, Nadi, and Nadroga/Navosa), 28 health centers and 24 nursing stations (11). The study included *S*. Typhi isolates cultured between January 2020 and December 2023. All laboratory procedures (culture and antimicrobial susceptibility testing) were performed in Lautoka hospital. Minimum inhibitory concentration (MIC) for ciprofloxacin was not performed prior to June 2023. Whole genome sequencing and additional broth microdilution phenotypic testing was performed at Microbiological Diagnostic Unit Public Health Laboratory in Melbourne, Australia. Chromosomal markers of antimicrobial resistance were predicted using the Typhi module (12) within Mykrobe (13). Details on the bioinformatics methods are provided in the Supplemental material.

**Fig 1:**
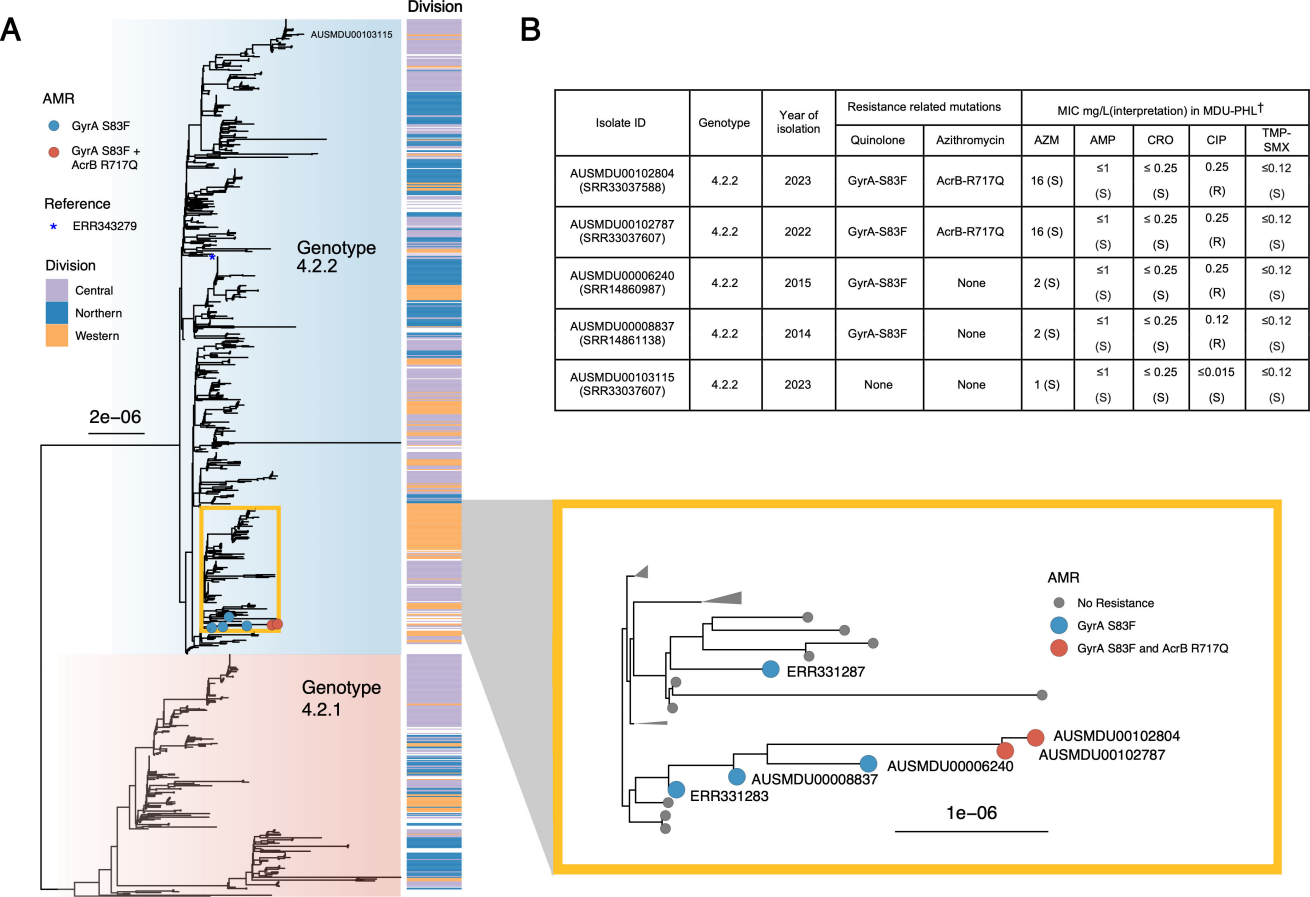
Population structure and phenotypic antimicrobial resistance profile of *S*. Typhi isolates in Fiji. (**A**) Maximum likelihood tree built from 578 parsimony informative SNPs of 978 genotype 4.2 *S*. Typhi isolates relative to the genotype 4.2.2 reference genome ERL072973 (position indicated by an asterisk). Shading refers to *S*. Typhi genotype as assigned by genotyphi. The associated health division where the isolate was isolated is displayed by the division column. The inset (yellow box) relates to the position of antimicrobial-resistant (AMR) isolates containing chromosomal point mutations in GyrA S83F (blue dots) and GyrA S83F plus AcrB R717Q (red dots). (**B**) Phenotypic antimicrobial susceptibility results of clinical *S*. Typhi isolates with GyrA (S83F) mutation conferring elevated MIC to ciprofloxacin (interpreted using EUCAST breakpoints) and secondary AcrB (R717Q) mutation conferring elevated MIC to azithromycin relative to a representative wild-type isolate (AUSMDU00103115/SRR33037607). †Broth microdilution using the Sensititre system in CMV5AGNF plate. MIC testing was done in December 2024 at the Microbiological Diagnostic Unit Public Health Laboratory. AZM, azithromycin; AMP, ampicillin; CRO, ceftriaxone; TMP-SMX, trimethoprim-sulfamethoxazole; MIC, minimum inhibitory concentration; MDU-PHL, Microbiological Diagnostic Unit Public Health Laboratory.

We sequenced 197 isolates from 176 patients, representing 38% of the total during the study period. Almost all isolates (99.5%) belonged to the genotype 4.2 with subclade 4.2.2 majority (84.3%). Two genetically related subclade 4.2.2 isolates (1 SNP difference) carried chromosomal point mutations in the quinolone resistance determining region of DNA gyrase at codon 83 (GyrA-S83F), a marker of ciprofloxacin resistance; and the AcrB efflux pump at codon 717 (AcrB-R717Q), a marker associated with resistance to azithromycin (14) (Fig. 1). Review of 781 historical Fijian isolates from Central and Northern divisions between 1981 and 2019 identified four additional isolates with the GyrA-S83F mutation and three likely evolved from an undefined single common ancestor (Fig. 1A). Four of these isolates were tested phenotypically and all exhibited resistance (MIC = 0.25 mg/L) to ciprofloxacin (Fig. 1B). While the two isolates with secondary AcrB-R717Q mutation had an MIC of 16 mg/L, they were higher compared with isolates without AcrB mutation (MIC = 1-2 mg/L). These two isolates in the current study were cultured from blood samples collected in December 2022 and February 2023 from Nadroga/Navosa and Ba subdivisions, respectively. Additional information on epidemiological linkage and treatment outcome is not available.

Azithromycin resistance in *S*. Typhi is uncommon (15). The first case was identified in Bangladesh among the MDR subclade of 4.3.1 in 2013 (14). More cases were subsequently reported in other countries with AcrB-R717Q/L mutations arising independently with no apparent epidemiological link (16–20). Similarly, the Fijian isolates with point mutations are genomically distinct and occurred in a non-MDR genotype. International importation is unlikely, as the 4.2 genotype is an archetypical *S*. Typhi genotype of Fiji (6, 7) and associated travelers from Fiji (8).

To conclude, we identified the emergence of MDR S. Typhi strains in the endemic 4.2 genotype. We recommend enhanced phenotypic surveillance with introduction of routine MIC testing for ciprofloxacin (21) and ongoing genomic surveillance to monitor the evolution of the current strains and detection of newly emerging AMR. Further investigation is warranted to demonstrate the correlation between AcrB-R717Q/L mutations, phenotypic azithromycin resistance, including MIC breakpoints and clinical outcomes. The use of typhoid conjugate vaccination (TCV) in the Northern Division has reduced disease burden (22); expansion of TCV will strengthen nationwide typhoid control.

## SUPPLEMENTAL MATERIAL

Supplemental material aac.00588-25-s0001.docx Bioinformatic methods.

ASM does not own the copyrights to Supplemental Material that may be linked to, or accessed through, an article. The authors have granted ASM a non-exclusive, world-wide license to publish the Supplemental Material files. Please contact the corresponding author directly for reuse.
