# Supplementary material for: Emergence of azithromycin and ciprofloxacin non-susceptible genotype 4.2.2 S. Typhi in Fiji
Source: Antimicrob Agents Chemother. 2025 Sep 22;69(11):e00588-25. doi: 10.1128/aac.00588-25 (PMC12587616; doi:10.1128/aac.00588-25)
Supplement: Supplemental material — Bioinformatic methods. [file aac.00588-25-s0001.docx]

**Supplementary methods**

Bioinformatic methods were adapted from Getahun Strobel et al, [1]. In brief**,** Illumina paired-end short reads were mapped to the Fijian *S*. Typhi reference strain ERL072973 (genotype 4.2.2, accession number LT904777.2) using snippy v4.6.0 (github/tseemann/snippy). Mobile genetic elements and genomic regions of irregular SNP density were previously identified in the reference genome and the isolate core genome alignment using Gubbins v2.4.1. SNPs present in >95% isolates (soft-core threshold) were kept in the final alignment using <https://github.com/rrwick/Core-SNP-filter> [2]. This resulted in a tree with 1309 soft-core SNPs of which 578 were parsimony-informative. The resulting consensus SNP alignment was used to build a maximum-likelihood tree with IQ-TREE v1.6.12 [3] with a GTR+F+G4 model. Genetic relatedness of individual AMR containing strains was determined by comparison of the variant call format files from snippy using vcftools. Genotyphi and point mutation resistance was predicted using the Typhi module [4] within Mykrobe v 0.11.0 [5].

**References**

1. Getahun Strobel A, Hayes AJ, Wirth W, et al. Genetic heterogeneity in the *Salmonella* Typhi Vi capsule locus: a population genomic study from Fiji. Microb Genom **2024**; 10(9).

2. Taouk ML, Featherstone LA, Taiaroa G, et al. Exploring SNP filtering strategies: the influence of strict vs soft core. Microb Genom **2025**; 11(1).

3. Nguyen LT, Schmidt HA, von Haeseler A, Minh BQ. IQ-TREE: a fast and effective stochastic algorithm for estimating maximum-likelihood phylogenies. Mol Biol Evol **2015**; 32(1): 268-74.

4. Danielle J. Ingle, Jane Hawkey, Martin Hunt, et al. Typhi Mykrobe: fast and accurate lineage identification and antimicrobial resistance genotyping directly from sequence reads for the typhoid fever agent *Salmonella* Typhi. bioRxiv **2024**; 09.30.613582. DOI: [10.1101/2024.09.30.613582](https://doi.org/10.1101/2024.09.30.613582)

5. Hunt M, Bradley P, Lapierre SG, et al. Antibiotic resistance prediction for Mycobacterium tuberculosis from genome sequence data with Mykrobe. Wellcome Open Res **2019**; 4: 191.
